# Reproducibility Assessment of Independent Component Analysis of Expression Ratios From DNA Microarrays

**DOI:** 10.1002/cfg.298

**Published:** 2003-06

**Authors:** David Philip Kreil, David J. C. MacKay

**Affiliations:** 1 Department of Genetics University of Cambridge Downing Street Cambridge CB2 3EH UK; 2 Inference Group Cavendish Laboratory Madingley Road Cambridge CB3 0HE UK

## Abstract

DNA microarrays allow the measurement of transcript abundances for thousands of genes in parallel. Most commonly, a particular sample of interest is studied next to a neutral control, examining relative changes (ratios). Independent component analysis (ICA) is a promising modern method for the analysis of such experiments. The condition of ICA algorithms can, however, depend on the characteristics of the data examined, making algorithm properties such as robustness specific to the given application domain. To address the lack of studies examining the robustness of ICA applied to microarray measurements, we report on the stability of variational Bayesian ICA in this domain. Microarray data are usually preprocessed and transformed. Hence we first examined alternative transforms and data selections for the smallest modelling reconstruction errors. Log-ratio data are reconstructed better than non-transformed ratio data by our linear model with a Gaussian error term. To compare ICA results we must allow for ICA invariance under rescaling and permutation of the extracted *signatures*, which hold the loadings of the original variables (gene transcript ratios) on particular latent variables. We introduced a method to optimally match corresponding signatures between sets of results. The stability of signatures was then examined after (1) repetition of the same analysis run with different random number generator seeds, and (2) repetition of the analysis with partial data sets. The effects of both dropping a proportion of the gene transcript ratios and dropping measurements for several samples have been studied. In summary, signatures with a high relative data power were very likely to be retained, resulting in an overall stability of the analyses. Our analysis of 63 yeast wildtype vs. wild-type experiments, moreover, yielded 10 reliably identified signatures, demonstrating that the variance observed is not just noise.


**Comparative and Functional Genomics**
*Comp Funct Genom* 2003; **4**: 300–317.
Published online in Wiley InterScience (www.interscience.wiley.com). **DOI:** 10.1002/cfg.298

**Primary Research Paper**

### Reproducibility assessment of independent component analysis of expression ratios from DNA microarrays

David Philip Kreil[1,2]* and David J. C. MacKay[2]
[1] *Department of Genetics, University of Cambridge, Downing Street, Cambridge CB2 3EH, UK*
[2] *Inference Group, Cavendish Laboratory, Madingley Road, Cambridge CB3 0HE, UK*

**Correspondence to:*
*David Philip Kreil, Department of Genetics, University of Cambridge, Downing Street, Cambridge CB2 3EH, UK.*
*E-mail: kreil@ebi.ac.uk*

Received: 6 November 2002
Revised: 30 March 2003
Accepted: 4 April 2003

### Abstract

**DNA microarrays allow the measurement of transcript abundances for thousands of genes in parallel. Most commonly, a particular sample of interest is studied next to a neutral control, examining relative changes (ratios). Independent component analysis (ICA) is a promising modern method for the analysis of such experiments. The condition of ICA algorithms can, however, depend on the characteristics of the data examined, making algorithm properties such as robustness specific to the given application domain. To address the lack of studies examining the robustness of ICA applied to microarray measurements, we report on the stability of variational Bayesian ICA in this domain. Microarray data are usually preprocessed and transformed. Hence we first examined alternative transforms and data selections for the smallest modelling reconstruction errors. Log-ratio data are reconstructed better than non-transformed ratio data by our linear model with a Gaussian error term. To compare ICA results we must allow for ICA invariance under rescaling and permutation of the extracted *signatures*, which hold the loadings of the original variables (gene transcript ratios) on particular latent variables. We introduced a method to optimally match corresponding signatures between sets of results. The stability of signatures was then examined after (1) repetition of the same analysis run with different random number generator seeds, and (2) repetition of the analysis with partial data sets. The effects of both dropping a proportion of the gene transcript ratios and dropping measurements for several samples have been studied. In summary, signatures with a high relative data power were very likely to be retained, resulting in an overall stability of the analyses. Our analysis of 63 yeast wild-type vs. wild-type experiments, moreover, yielded 10 reliably identified signatures, demonstrating that the variance observed is not just noise. Copyright © 2003 John Wiley & Sons, Ltd.**

**Keywords: independent component analysis; expression ratios; microarray data; ensemble learning**

### Introduction

DNA microarrays allow the simultaneous measurement of transcript levels for thousands of genes (Schena *et al*., 1995). These give an indication of which genes have been turned on in a given sample. Using multiple 'channels' per measurement, several transcript levels are usually determined for each gene. Most commonly, one channel is used for a neutral control and one other channel measures the transcript level of a particular sample under investigation. It is then typical to focus on the channel ratios. Samples can be from different tissue types, developmental stages, disease classes or specifically genetically engineered organisms.

Copyright © 2003 John Wiley & Sons, Ltd.

Independent component analysis (ICA) is a promising modern method for the analysis of such experiments. More traditionally, ICA has been used for blind source separation of noisy images, sound recordings and electrophysiological data. Only recent studies have applied ICA to microarray experiments (Miskin, 2001; Liebermeister, 2002; Lin *et al.*, 2002). ICA can be seen as a less constrained extension of other factor analyses, e.g. the popular principal components analysis. See Bell and Sejnowski, 1995; MacKay, 1996; Pearlmutter and Parra, 1996; Makeig *et al.*, 1997; Roberts and Everson, 2001; Hyvarinen *et al.*, 2001 for a general discussion of ICA and typical earlier applications in other domains.

ICA relies on a non-Gaussian distribution of the underlying latent variables that are to be uncovered. As a consequence, certain properties of algorithms for ICA can depend on characteristics of the data examined, which will be different according to the application domain of the analysis.

For microarray data, little is known about the expected distributions of the underlying latent variables. Moreover, in contrast to the very high number of measurement variables ($\sim 10^4$, scaling with the number of genes examined), an everyday data set will consist of only a few samples ($\sim 10-100$). Testing results from applications of ICA in this domain for robustness is therefore of great interest.

Since it is common in the field to transform or preprocess the data, we first examined alternative transforms and data selections for the smallest modelling reconstruction errors.

A test for robustness requires comparing ICA results, e.g. the *signatures*, which hold the loadings of the original variables (gene transcript ratios) on particular latent variables. ICA is invariant under rescaling and permutation of these signatures and any comparison of two sets of signatures must allow for that. To this end, we introduced a method to optimally match corresponding signatures from two sets to one another.

**Figure 1.** Iterative proportional fitting, algorithm overview. The procedure aborts after a specified maximum number of iterations. A faster converging, yet possibly less robust, alternative is obtained by initializing `M = S` and updating `M = M ** mu`, where ** stands for element-wise exponentiation. `update_row_scaling` rescales individual rows so that they each sum to 1

Copyright © 2003 John Wiley & Sons, Ltd.

```

function analyse_similarity_matrix(S):

  ok   = zeros;

  good = zeros;

  M = S;

  row_index=0;

  repeat

    i = highest_diagonal_element_index_next(row_index,M);

    if M(i,i) >= max(row_of(M,i)) and

       M(i,i) >= max(column_of(M,i)) and

       M(i,i) >= 0.1 then

      ok(i) = 1;

      if M(i,i) >= 0.6 then

        good(i) = 1;

      end_if

      row_of(M,i) = zeros;

      column_of(M,i) = zeros;

    end_if

  until no_changes_to_M; return(ok,good,S);

```

**Figure 2.** Similarity matrix analysis, algorithm overview. If a diagonal element similarity score is less than 0.1, it is considered too bad for a match. We consequently call similarity scores of at least 0.1 'ok' matches. Matches meeting the arbitrary threshold score of 0.6 are called 'good'. `highest_diagonal_element_index_next` will, in successive calls, return all row indices of its matrix argument so that the corresponding diagonal element values decrease. Once the set of all row indices has been exhausted, the function will restart with the index for the highest such element. By allowing multiple iterations, clear cases can be dealt with first, removing ambiguities for matches with weaker similarity score. The loop is aborted after a specified maximum number of iterations

We then examined the stability of signatures after (1) repetition of the same analysis run with different random number generator seeds, and (2) repetition of the analysis with partial data sets. We studied both the effects of dropping a proportion of the gene transcript ratios and dropping measurements for a fraction of the samples.

### Methods

### Selection and preprocessing of data sets

We used data published by Hughes *et al.* (2000), who provide one of the most extensive data sets publicly available, and moreover performed 63 'neutral' vs. 'neutral' experiments, examining the natural variation seen in unaltered wild-type yeast. It is this subset of 63 samples that this study uses.

The data were first preprocessed to remove all the ratios of genes for which there occurred values that were not a number, or infinite. Data for 1464 such genes were dropped, reducing the size of the final data matrix to $63 \times 4870$. All further analysis was done on this subset of the data.

The data, as provided by Rosetta Inc. (Hughes *et al.*, 2000), contain $\log_{10}$-transformed channel ratios and estimates of their experimental errors. For an examination of non-transformed ratios, both the data and the experimental error estimates have been appropriately reverse-transformed.

### Algorithms

ICA was performed using ensemble learning (reviewed by MacKay, 1995) as implemented in MatLab by Miskin (2001). With $s$ enumerating samples (e.g. different tissues or experimental conditions) and $i$ enumerating the original input variables (gene transcript ratios), we get a decomposition:

$$D_{si} = \sum_{l} A_{sl} B_{li} + \nu_{si} \quad (1)$$

Copyright © 2003 John Wiley & Sons, Ltd. 

where $l$ enumerates the latent variables, and $\nu$ allows for Gaussian noise. The matrix $B = (\sigma_l)$ holds the *signatures*: a signature $\sigma_l = (\sigma_{li})_i$ shows how much each of the original input variables contributes to a particular latent variable; $\sigma_{li}$ is often also called the *loading* of gene $i$ on component $l$. The amounts required to reconstruct the data in latent variable space are given by the so-called mixing matrix, $A$. For any particular $A$ and $B$, $\nu = D - AB$ gives the reconstruction error, i.e. the magnitudes of noise required for the model to match the observed data.

In contrast to other approaches to ICA, which calculate a point estimate of the parameters of the model, ensemble learning computes a factorization approximation to the full posterior (MacKay, 1995). The hyperparameters of this approximate posterior are initialized with random values.

Signatures from two ICA runs were compared to one another using an iterative proportional fitting (IPF) procedure to obtain a best-guess permutation matrix giving the required reordering for an optimal match. For two signatures, $\sigma$ and $\tau$, a similarity measure:

$$s_{\sigma\tau} = \frac{\left(\sum \sigma_i \tau_i\right)^2}{\sum \sigma_i^2 \sum \tau_i^2} \qquad (2)$$

was defined, which is invariant under rescaling of the signatures. Here, the sums run over all original variables (gene transcript ratios). Figure 1 outlines the IPF procedure; further details can be found in the online supplement.

To rank latent variables according to their contribution to the reconstruction, we calculate the relative data power for a latent variable:

$$p_l = \frac{\sum_{s,i} (a_{sl} b_{li})^2}{\sum_{s,i} d_{si}^2} = \frac{\sum_s a_{sl}^2 \sum_i b_{li}^2}{\sum_{s,i} d_{si}^2} \qquad (3)$$

Note that this quantity can also become larger than 1 for certain values of $\nu$.

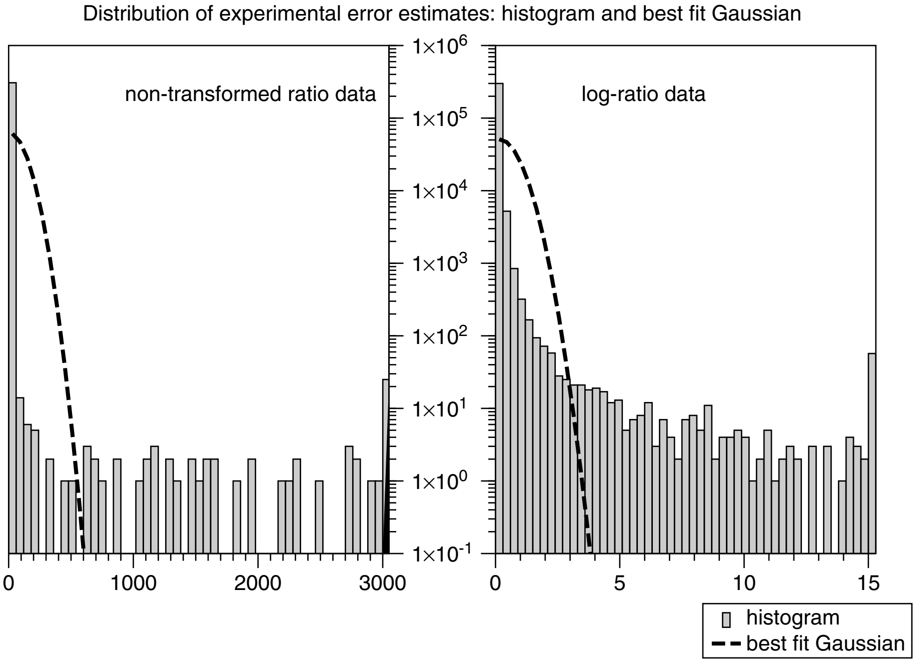

**Figure 3.** Distribution of experimental error estimates for (non-transformed) ratio data (left) and log-ratio data (right). The horizontal axes correspond to the magnitudes of the experimental error estimates, the heights of the bars to their frequencies. Note that the presentation is semi-logarithmic. The distributions of errors show heavy tails with a wide range of observed values in the data set. In the above histograms, moreover, all error values larger than a cut-off have been combined into the last bin. For the left-hand histogram, the cut-off was 3000; the full range of the error values was ~0.4 ... 38 543 (not shown). For the right-hand histogram, the cut-off was 15; the full range of the error values was ~0.09 ... 167 (not shown)

Copyright © 2003 John Wiley & Sons, Ltd. 

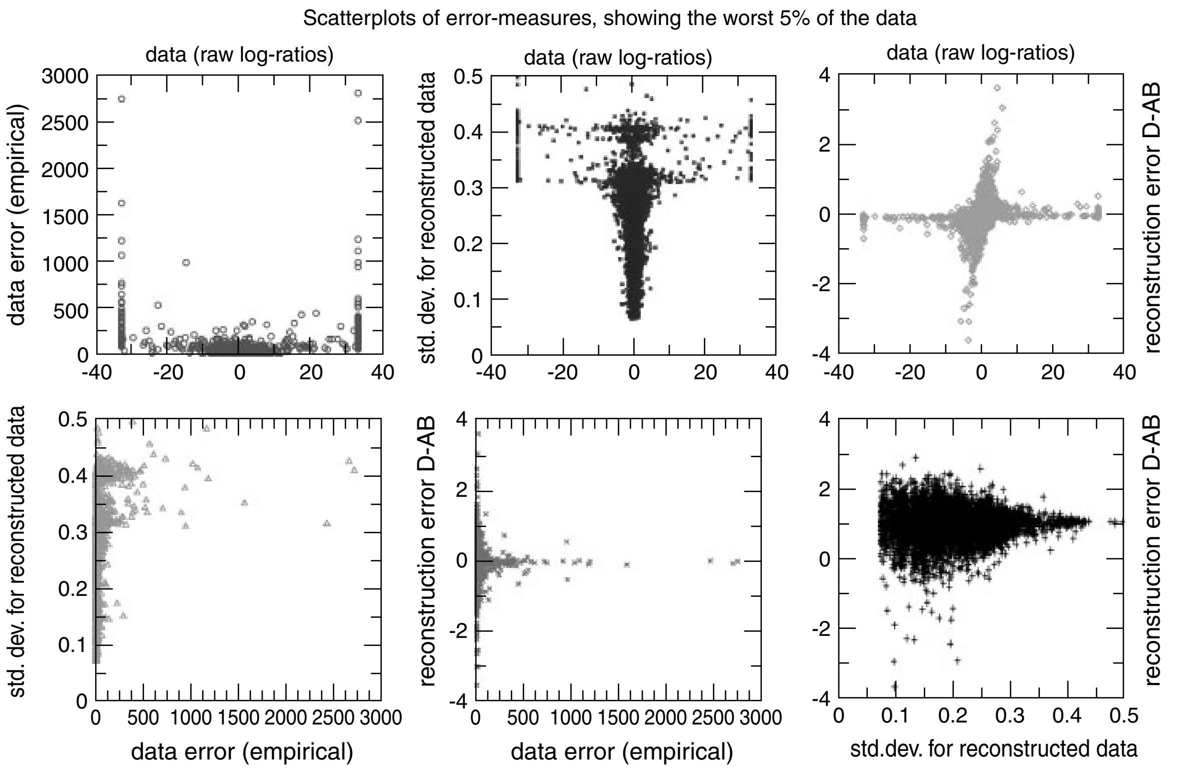

**Figure 4.** Scatterplots of error measures for log-ratios, showing the worst 5% of the data. The graphs plot the relationships between various error measures and the log-ratio data. The error measures shown are: experimental error estimates (empirical data errors), standard deviations for the reconstructed data from ensemble learning, and reconstruction errors (original minus data reconstructed from the model)

Pairs of ICA results were then compared using the heuristic described in Figure 2. A pair of signatures is considered to be matching one another when no better score could have been obtained by pairing any one of them with a different signature available in the respective other set. Matching signatures are not reconsidered in further rounds of this test, and signatures of high relative data power are examined first. This effectively allows more ambiguous cases to also be resolved.

### Study details

The full analysis was first repeated with 10 different random number generator seeds, yielding $\binom{10}{2} = 45$ pairwise comparisons. In the following other tests, to separate the effect caused by a changed random number generator seed, analyses were all run after using the same seed to initialize the random number generator (RNG).

Six independent subsets of the complete data were then each generated by dropping data for a different random selection of 974 genes (20%). The obtained signatures were compared to the signatures of the full analysis. Four independent subsets of the complete data were generated by

**Table 1.** Outline of experiments testing reproducibility

| *N* | *pw* | Type |
|---|---|---|
| 10 | 45 | RNG seed variation |
| 6 | 6 | Dropping a random 5% of the original variables |
| 6 | 6 | Dropping a random 20% of the original variables |
| 6 | 6 | Dropping a random 35% of the original variables |
| 6 | 6 | Dropping a random 50% of the original variables |
| 4 | 6 | Dropping a random 5% of measurement samples |
| 4 | 6 | Dropping a random 20% of measurement samples |
| 4 | 6 | Dropping a random 35% of measurement samples |
| 4 | 6 | Dropping a random 50% of measurement samples |

The table shows the type of experiment, how many independent data sets have been generated (*N*), and the number of pairwise comparisons made (*pw*). Note that when original variables have been dropped, comparisons have been to results from a complete reference set.

Copyright © 2003 John Wiley & Sons, Ltd. 

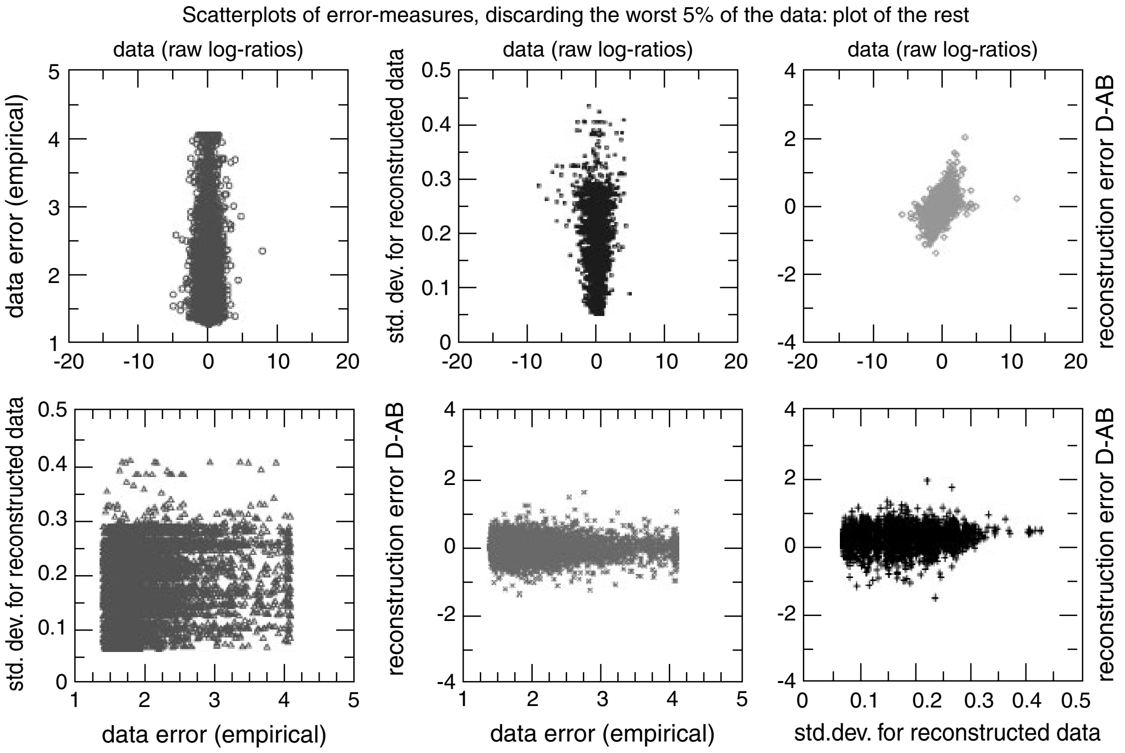

**Figure 5.** Scatterplots of error measures for log-ratios, excluding the worst 5% of the data. A random 5% subset of the remaining data has been plotted. The scales of the plots show the ranges of the entire remaining data. See also caption to Figure 4

**Table 2.** Standard deviations about zero of the reconstruction error for data transform alternatives

| (a) | Model | Ratios | Log-ratios | (b) | Model | Ratios | Log-ratios |
|---|---|---|---|---|---|---|---|
| | Ratio scale | 0.039 | 0.028 | | Ratio scale | 0.38 | 0.03 |
| | Log-ratio scale | 0.025 | 0.012 | | Log-ratio scale | 2.42 | 1.72 |

After application of the ICA model to the (non-transformed) ratio data, or to log-ratios, the standard deviations about zero of the reconstruction error have been determined in both scales (log and non-transformed). For the relative errors, only non-zero data points have been considered. a, absolute reconstruction error; b, relative reconstruction error.

dropping a different random selection of 13 samples (20%). The four signatures obtained were matched to one another, again yielding six comparisons. The experiments dropping certain original variables or measurements were also done dropping 5%, 35% and 50% of the data, respectively (see Table 1).

After optimal reordering according to the permutation matrix constructed using IPF, the similarity measure (2) was calculated for all signature pairs, and analysed as to whether individual signatures could be matched up. We visualized the resulting similarity matrices using Hinton-like plots: the area of a square corresponds to the size of the value represented; since all values are positive, black colour does *not* denote negative quantities, but is used to mark uniquely identified matches.

This algorithm was implemented in MatLab.

### Results

### Alternative data selection and transforms

Often, log-ratios are analysed rather than non-transformed ratios. We have examined reconstruction

Copyright © 2003 John Wiley & Sons, Ltd. 

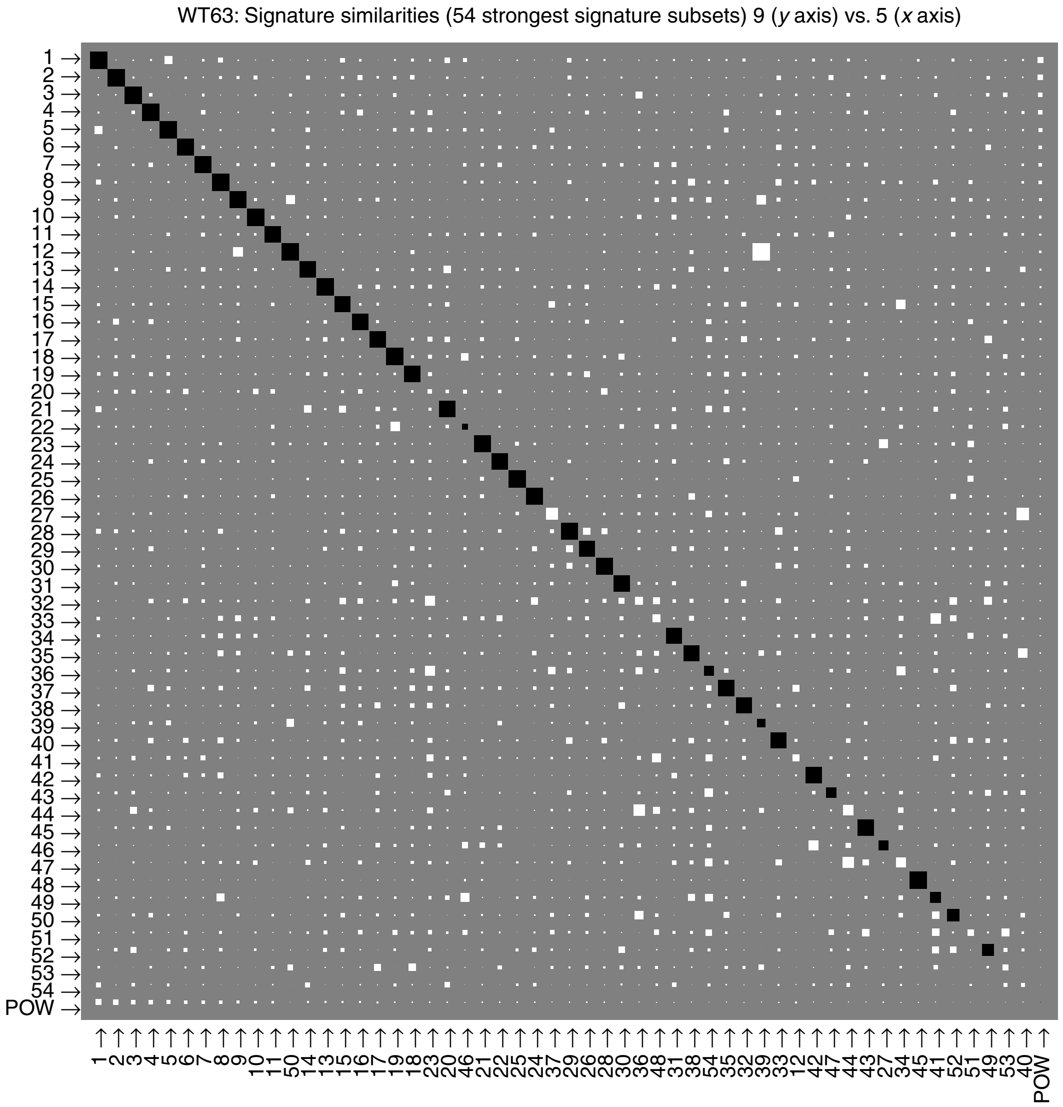

**Figure 6.** Hinton-like plot of the similarity matrix for sets of signatures obtained from analysis of the full data sets with different random number generator seeds. The area of the white blocks represents the values of the corresponding matrix entries, which can range from zero (no similarity) to one (perfect match). For a perfect match, two signatures must be co-linear. For reference, the relative data power (POW) is also displayed. Diagonal elements are coloured black if the match can be considered optimal by heuristic criteria (see Methods)

errors for both alternatives. It is further customary in the field to normalize data with respect to the experimental error estimates for each data point. We prefer to explicitly model data and errors, using the non-normalized data directly. It has been observed before that the distribution of reconstruction errors has heavy tails (Miskin, 2001) compared to a Gaussian distribution. This is also true for the experimental error estimates in our data set (Figure 3).

A Gaussian distribution would, however, be a useful approximation for reasonably compact error distributions. Plotting error measures vs. data values for log-transformed ratios (Figures 4 and 5) and non-transformed ratios (see online supplement) shows that by exclusion of the data points with the worst 5% of experimental error estimates, one can indeed get a compact range of errors, avoiding more complex error models in a first approximation. The plots also suggest that, in the Rosetta

Copyright © 2003 John Wiley & Sons, Ltd. 

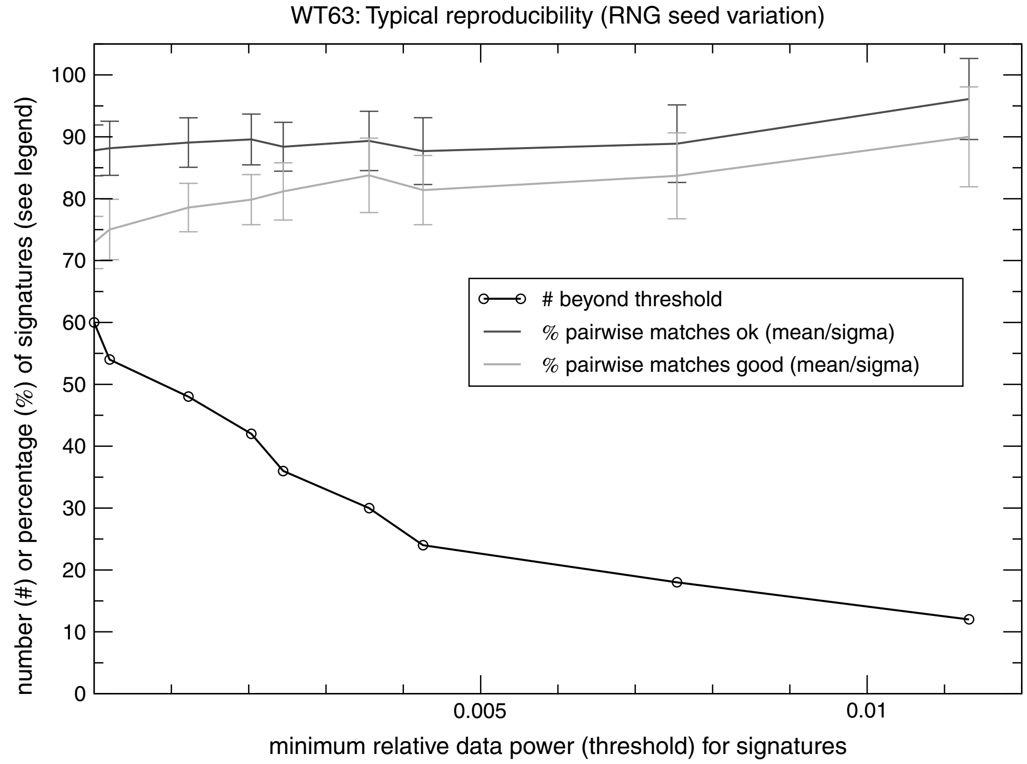

**Figure 7.** Pairwise identification percentages for different random number generator seeds. For 45 attempts of pairwise matching of signatures from otherwise identical ICA runs with different random number generator seeds, this plot shows how many signatures could be identified with a similarity score of at least 0.1 (ok) and how many also had a good similarity score of 0.6 or higher. Signature sets were restricted by different thresholds of relative data power before the comparison (shown on the *x* axis). For each such threshold, the graph shows the means and error bars (standard deviations) of the percentages of the signatures that could be identified in the 45 pairwise comparisons. The number of signatures above the threshold is also displayed (circles; the total number, not the percentage, is plotted)

data set, several data points with particularly high errors have actually been generated by range truncation: extreme values appear to have been set to the finite values $\pm 2$ on the log-scale. Clearly, we want to exclude these from a quantitative model. All further results have been obtained after pruning the data points with the worst 5% of experimental error estimates.

Comparing the distributions of the reconstruction errors for models of log-ratios and non-transformed ratios, one could see that the log-ratio model had the lower reconstruction errors both in log-ratio space and in non-transformed ratio space (Table 2). A linear model in log-ratio space corresponds to multiplicative effects for the non-transformed ratios. Such effects have been proposed for gene expression that is regulated by multiple transcription factors (Bussemaker *et al.*, 2001). There is also an alternative explanation for the lower reconstruction errors in log-ratio space: the log-transform makes the range of errors more compact and easier to model with a Gaussian error distribution. All further results have been obtained from log-ratio data.

### Reproducibility

In each study, the repeated analyses yielded pairwise similarity matrices which were fairly alike. Only examples and summaries may therefore be shown.

### Effects of different random number generator seeds

Figure 6 shows a similarity matrix obtained from the analysis of the full data set with different random number generator seeds. Out of the 10 runs, a pair of signature sets that yielded one of the

Copyright © 2003 John Wiley & Sons, Ltd. 

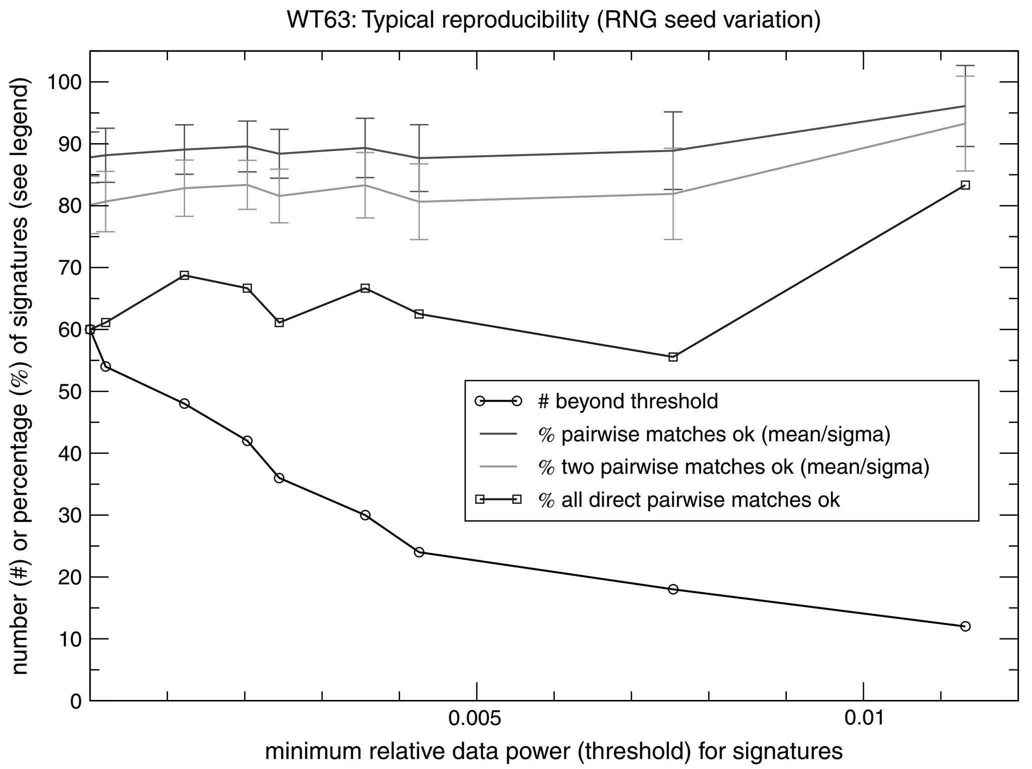

**Figure 8.** Identification percentages for different random number generator seeds. For 45 attempts of pairwise matching of signatures from otherwise identical ICA runs with different random number generator seeds, two pairs of matched sets have always been combined. This means that this plot displays how many signatures could be identified in at least two comparisons in addition to results from simple pairwise comparisons. Another curve shows how many signatures from an arbitrarily picked set could be identified in all the pairwise matches to the remaining nine sets (boxes). Signature sets were restricted by different thresholds of relative data power before the comparison. For each such threshold, the graph shows the means and error bars (standard deviations) of the percentages of the signatures that could be identified. The number of signatures above the threshold is also displayed (circles; the total number, not the percentage is plotted)

worst matches has been chosen for this example. Only latent variables with a relative data power of at least $2 \times 10^{-4}$ have been included, leaving 54 of the original 63. The high similarity scores along the diagonal show good pairwise matches, while occasional large off-diagonal elements indicate similarity to other signatures than the assigned optimal match.

Examining all 45 pairwise matches, we found that for $(88 \pm 4)\%$ of the signatures a best pairwise match can be identified (range 78–96%), most of which have a 'good' score of 0.6 or higher [$(75 \pm 5)\%$, range 67–85%].

One wonders whether exclusion of latent variables with low relative data power could improve the picture. Indeed, using a cutoff value of $\sim$1%, even $(96 \pm 7)\%$ of the remaining signatures can be matched pairwise (range 90–100%), most of which have a 'good' score [$(90 \pm 8)\%$, range 82–100%]. Increasing the cutoff above that does not further help these statistics: only a handful of signatures remain. If only a few of these are reversed in their order by relative data power in a pair of compared sets, so that a signature is amongst the $N$ signatures of highest data power to be considered in one set, but not its matching partner in the other, no match can be found and a bad score results (e.g. at a cutoff value of 2%, one mismatch in a set of nine already gives a penalty of more than 10%). For example, a threshold of 6.5% for relative data power leaves only three signatures in set 1, and four from set 4. To perform IPF, the three signatures with the highest relative data power have to be chosen from each set. This, however, discards signature 4 from set 4, which is the best match to signature 3 of set 1 (data not shown). Figure 7 displays how well signatures

Copyright © 2003 John Wiley & Sons, Ltd. 

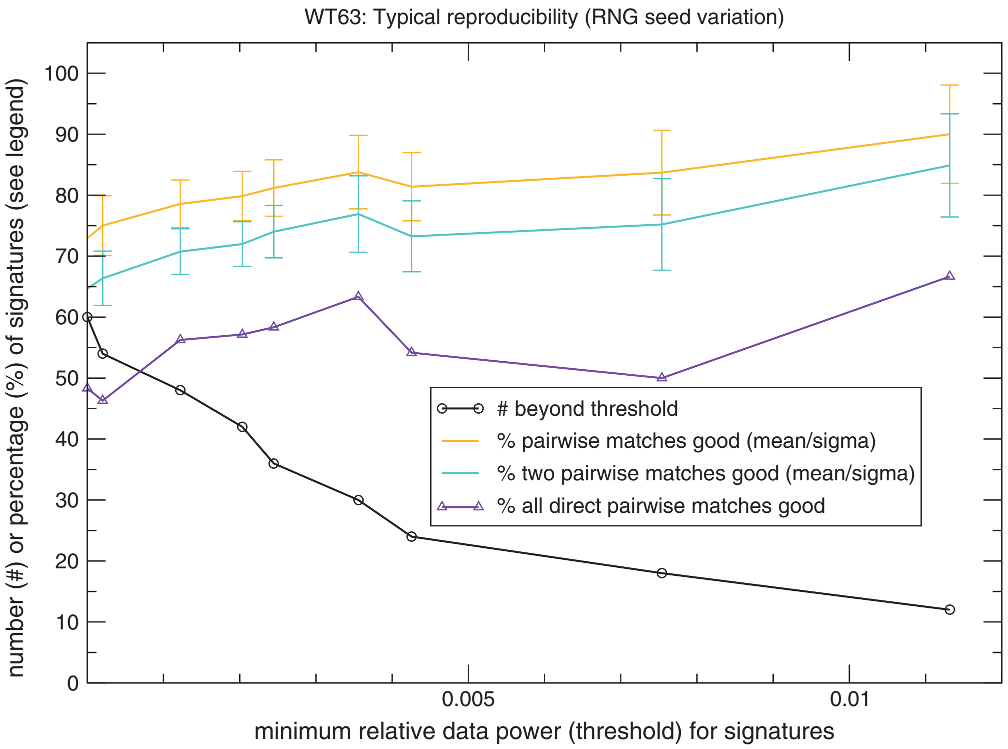

**Figure 9.** Identification percentages for different random number generator seeds, good matches. This figure shows results corresponding to those plotted in Figure 8. Here, only matches with a good similarity score were being counted

**Table 3.** Set 1 signatures identified in all pairwise matches to signature sets obtained with different random number generator seeds, for different thresholds

| Cutoff | *N* | Identified signatures with good (ok) similarity scores |
|---|---|---|
| $1.2 \times 10^{-37}$ | 60 | 1 2 4 5 6 7 8 9 10 11 12 (14) 17 18 19 20 21 22 24 25 26 27 29 30 32 33 (34) (36) (40) (41) 42 43 47 (51) 57 (58) |
| $2.0 \times 10^{-4}$ | 54 | 1 (3) 4 5 6 7 8 10 11 12 (14) (15) (16) 17 19 20 22 23 24 26 27 28 29 30 32 33 (34) (36) (41) 42 43 (44) 47 |
| $1.2 \times 10^{-3}$ | 48 | 1 2 (3) 4 5 6 7 8 9 10 11 12 (15) (16) 17 18 19 20 22 23 24 25 26 27 28 29 30 32 (36) (41) 42 43 (44) |
| $2.0 \times 10^{-3}$ | 42 | 1 2 (3) 4 5 6 7 8 9 10 11 12 17 18 19 20 (21) 23 24 25 26 27 28 29 30 32 (36) (41) |
| $2.5 \times 10^{-3}$ | 36 | 1 2 (3) 4 5 7 8 9 10 11 12 17 18 19 20 23 24 25 26 27 28 29 |
| $3.6 \times 10^{-3}$ | 30 | 1 2 3 4 5 (6) 7 9 10 11 12 17 18 19 20 23 24 25 27 28 |
| $4.3 \times 10^{-3}$ | 24 | 2 3 4 (6) 7 8 9 10 11 12 17 18 19 (20) 23 |
| $7.5 \times 10^{-3}$ | 18 | 1 3 4 (6) 7 8 9 10 12 17 |
| $1.1 \times 10^{-2}$ | 12 | 1 2 3 4 5 (6) 7 8 9 (11) |

The signatures of an arbitrarily picked set of ICA results (set 1) were determined, which could be identified in all the pairwise comparisons to the signature sets obtained from ICA with different random number generator seeds. This table lists these signatures for various relative data power thresholds, and also shows which of them had a good similarity score ($\geq$0.6). *N* gives the total number of signatures in the sets that were compared, i.e. this number of signatures were above the cutoff for all sets.

Copyright © 2003 John Wiley & Sons, Ltd. 

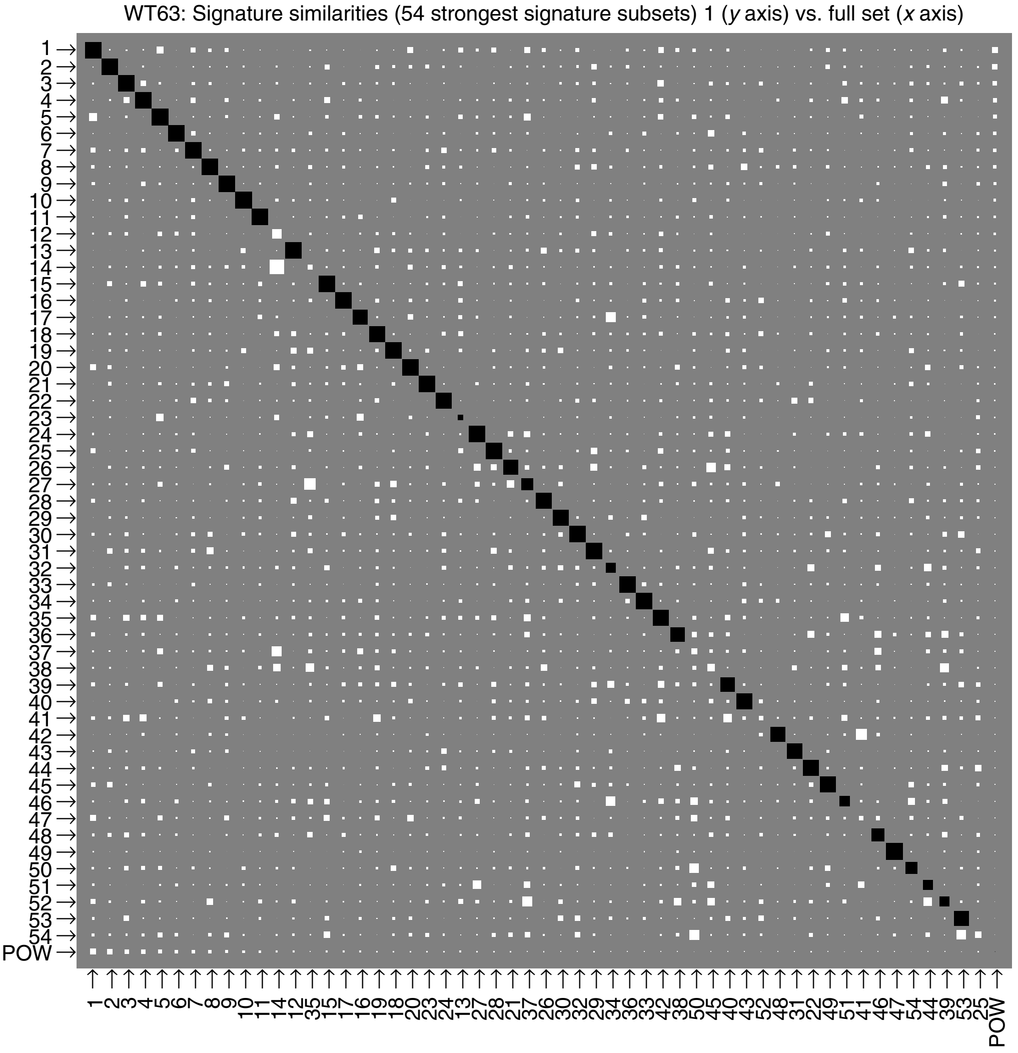

**Figure 10.** Hinton-like plot of the similarity matrix for ICA results on an 80% subset of variables, matched to the reference of the set of signatures obtained from analysis of the full data set (see caption to Figure 6). NB: For clarity of demonstration, the worst match from the six comparisons was chosen for this figure could be matched pairwise between sets for various thresholds.

Examination of which signatures could be identified in a pairwise match also showed that, for small or heterogeneous sets, this varies non-trivially with the choice of the threshold parameter: whether signatures with extreme loadings on particular original variables (gene transcript ratios) are included affects the subsequent normalization of this variable.

Having multiple pairwise comparison available, the question arises whether the same latent variables could stably be identified in each of the pairwise matches. Examining how many signatures of a given set could be identified in pairwise matches to at least two other sets yields, in the worst case, a reduction of 8% (see Figure 8). For an arbitrarily picked set 1, we also determined which signatures could be identified in all the pairwise comparisons to the remaining nine sets. In the worst case (for a cutoff value of $2 \times 10^{-4}$), this gave a reduction of more than 40%. For higher cutoff values (>0.5%), however, no further reduction was seen (data not shown). For a minimum relative data power threshold of $\sim$1%, in all sets, more than 90% of the signatures

Copyright © 2003 John Wiley & Sons, Ltd. 

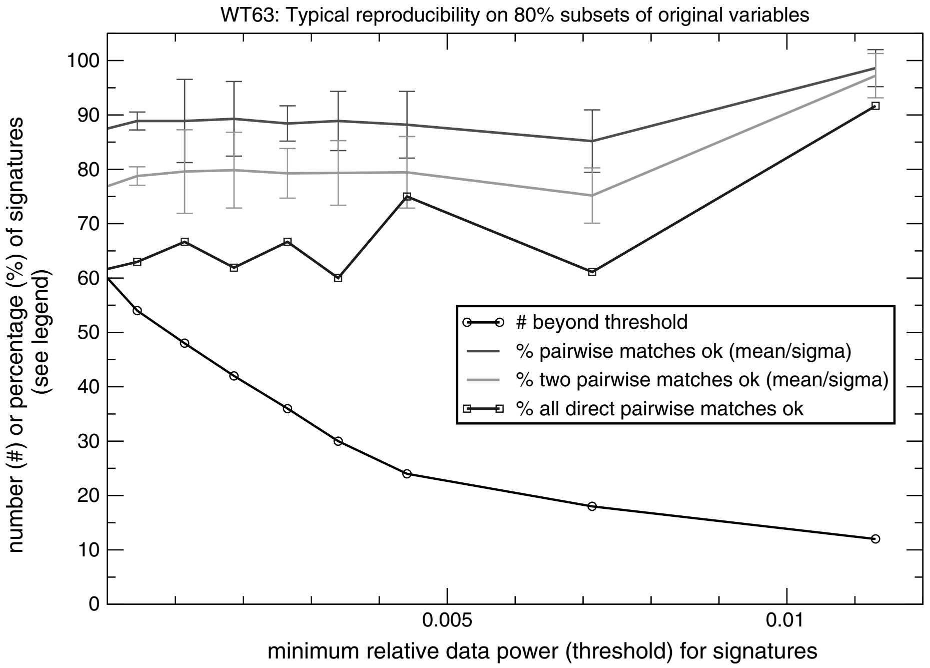

**Figure 11.** Identification percentages for results from different original variable subsets. For six attempts of pairwise matching of signatures from ICA of the entire data set (the reference) to results of ICA runs on different 80%-subsets of the original input variables, two pairs of matched sets have always been combined. This means that this plot shows how many signatures could be identified in at least two comparisons, in addition to results from simple pairwise comparisons. Another curve shows how many signatures of the reference set could be identified in all the six pairwise matches (boxes). (Also see caption to Figure 8)

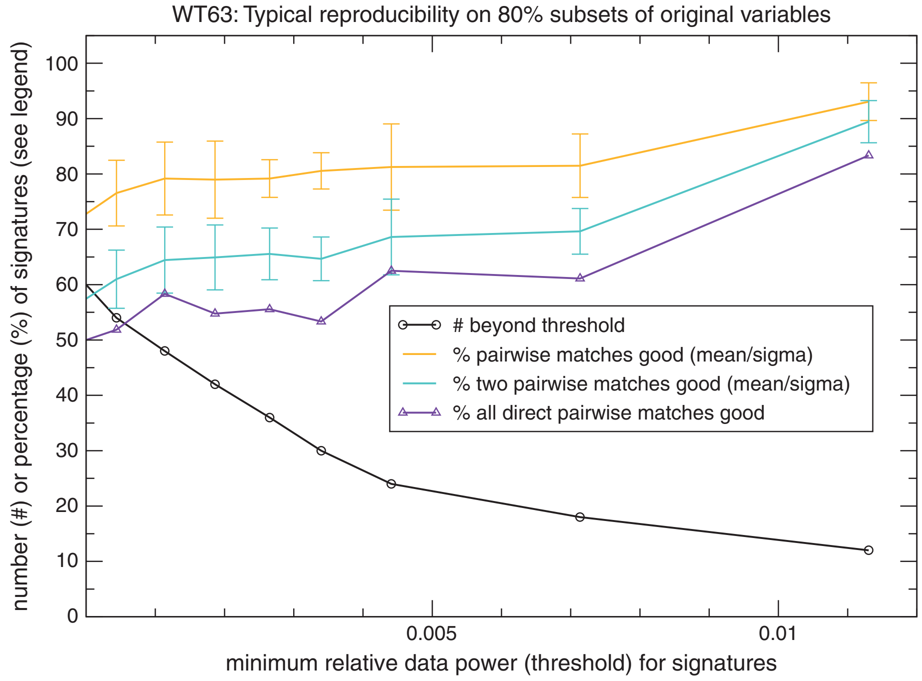

**Figure 12.** Identification percentages for results from different original variable subsets, good matches. This figure shows results corresponding to those plotted in Figure 11. Here, only matches with a good similarity score were being counted

Copyright © 2003 John Wiley & Sons, Ltd. 

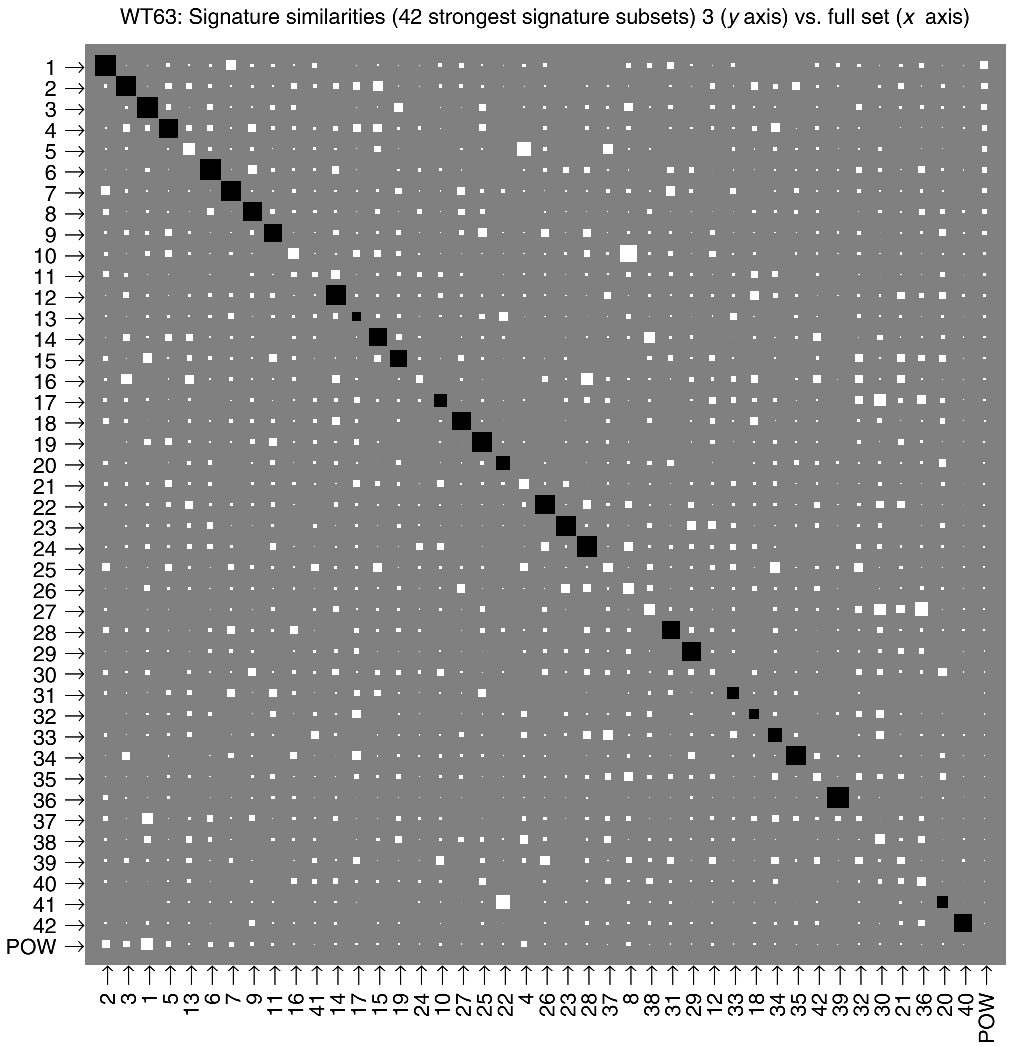

**Figure 13.** Hinton-like plot of the similarity matrix for ICA results on two different 80% subsets of measurement samples (see caption to Figure 6). NB: The worst match from the six comparisons was chosen for this figure could be identified in at least two other sets. Also, more than 80% of the signatures from set 1 were identified in all pairwise matches to the other nine sets.

Similarly for identified matches with a 'good' score (see Figure 9): A combination of two pairwise matches yielded a reduction of 9% compared to plain pairwise scores. The combination of all pairwise matches for set 1 gave a reduction of ∼15%. For a minimum relative data power threshold of ∼1%, in all sets, 85% of the signatures could be identified in at least two other sets with a good score ($\geq$0.6). Moreover, two-thirds of the signatures from set 1 were identified in all pairwise matches to the other nine sets with a good score.

The apparent irregularity of the curves showing the number of signatures found in all pairwise comparisons reflects that this quantity is derived for a single set, set 1, while the other plots average the results for multiple sets.

It was also interesting to check which particular signatures of set 1 could be identified, as the relative data power threshold was varied. From Table 3 one can see that not only are the lists of signatures at higher cutoffs almost subsets of those for lower cutoffs but that, moreover, for a high enough cutoff (∼1%) the list of identified

Copyright © 2003 John Wiley & Sons, Ltd. 

**Table 4.** Reference set signatures identified in all pairwise matches to signature sets obtained after deleting 20% of the variables, for different thresholds

| Cutoff | *N* | Identified signatures with good (ok) similarity scores |
|---|---|---|
| $1.2 \times 10^{-37}$ | 60 | 1 2 3 4 5 6 7 8 9 10 11 12 17 18 19 (20) 21 23 24 26 27 28 29 30 32 33 (38) (39) (42) 43 (46) 47 49 (53) (54) 57 58 |
| $2.0 \times 10^{-4}$ | 54 | 1 2 3 4 5 6 7 8 9 10 11 12 17 18 19 (20) 21 24 26 27 28 29 30 32 33 36 (38) (39) (40) 43 (46) 47 49 (51) |
| $1.2 \times 10^{-3}$ | 48 | 1 2 3 4 5 6 7 8 9 10 11 12 17 18 19 (20) 21 22 23 24 26 27 28 29 30 32 33 (39) (41) 42 43 (46) |
| $2.0 \times 10^{-3}$ | 42 | 1 2 3 4 5 6 7 8 9 10 11 12 (15) 17 18 19 (20) 23 24 26 27 28 30 32 33 (39) |
| $2.5 \times 10^{-3}$ | 36 | 1 2 3 4 6 7 9 10 11 12 (14) 17 18 (21) (22) 23 24 26 27 28 29 30 32 (35) |
| $3.6 \times 10^{-3}$ | 30 | 1 2 3 4 7 10 11 12 (14) 17 18 19 (20) 23 24 26 27 28 |
| $4.3 \times 10^{-3}$ | 24 | 1 2 3 4 6 7 8 9 10 11 12 (15) (16) 17 18 19 (20) 23 |
| $7.5 \times 10^{-3}$ | 18 | 1 2 3 4 6 7 8 9 10 11 12 |
| $1.1 \times 10^{-2}$ | 12 | 1 2 3 4 5 6 7 8 9 10 (11) |

Compared to a reference set calculated using the entire data, the signatures were determined which could be identified in *all* the pairwise comparisons to signatures obtained after deleting 20% of the original variables. The table lists these signatures for various relative data power thresholds, and also shows which of them had a good similarity score ($\geq$0.6). *N* gives the total number of signatures in the sets that were compared, i.e. this number of signatures were above the cutoff for all sets.

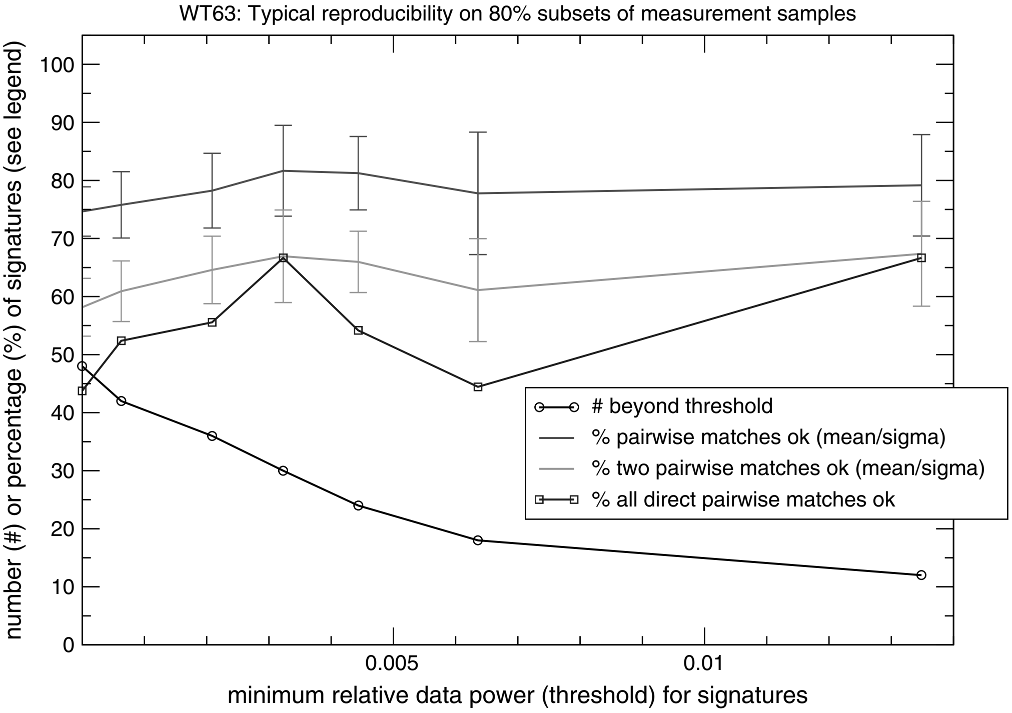

**Figure 14.** Identification percentages for results from different measurement sample subsets. For six attempts of pairwise matching of signatures from ICA of different 80% measurement sample subsets, two pairs of matched sets have always been combined. This means that this plot shows how many signatures could be identified in at least two comparisons in addition to results from simple pairwise comparisons. Another curve shows how many signatures from an arbitrarily picked set could be identified in all the pairwise matches to the remaining three sets (boxes). (Also see caption to Figure 8)

Copyright © 2003 John Wiley & Sons, Ltd. 

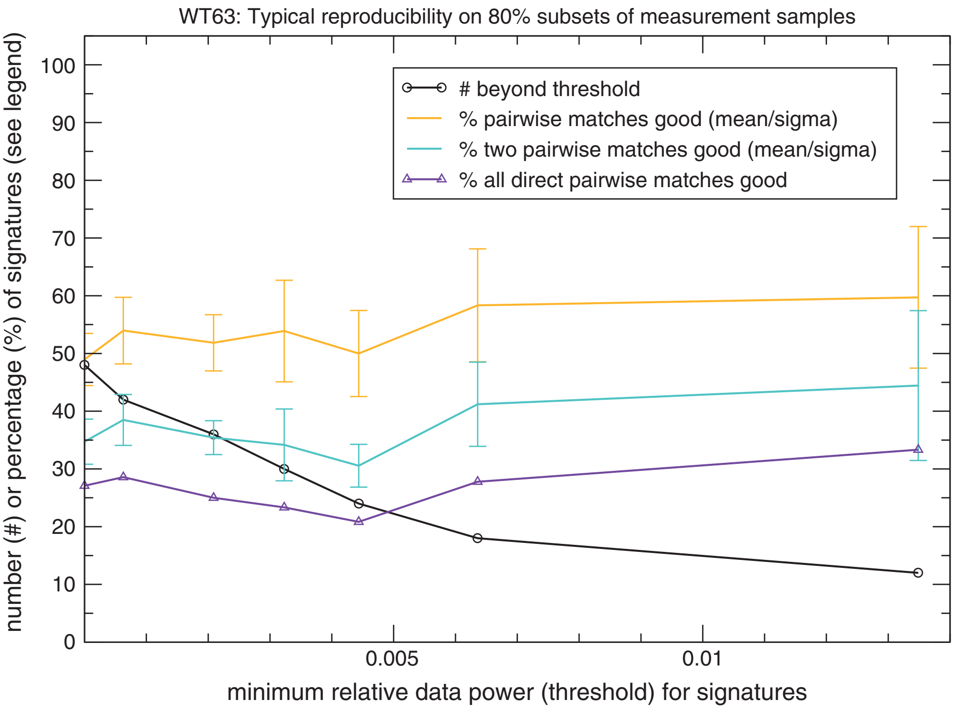

**Figure 15.** Identification percentages for results from different measurement sample subsets, good matches. This figure shows results corresponding to those plotted in Figure 14. Here, only matches with a good similarity score were being counted signatures is contiguous, starting with the signature of highest relative data power.

For practical purposes of assessing the reliability of a *single* set of signatures from this experiment, one could therefore pick a threshold of $\approx$1.5%. The higher the relative data power of a signature, the more likely it is to be reproducible. Only occasionally, the closest match to a signature of high relative data power in reruns of the analysis occurs with a much lower relative data power value. A second (verification) run is likely to expose such cases and may hence be advisable.

### Effects of excluding random subsets of the original variables

Comparison of signatures obtained after exclusion of a random 20% of the original input variables to those signatures from ICA of the full (reference) data set gives a similarly robust picture (Figures 10, 11 and 12). Although, again, there is some variation by cutoff value, a threshold of $\sim$1% will give signatures that remain identifiable after exclusion of a random 20% of the original variables. At the 1% threshold, over 90% of signatures could be identified in all six pairwise matches, most of which were good. In general, the higher the relative data power of a signature, the more likely it is to be robust. Since the lists compared in Table 4 show signatures that could be identified in all six pairwise matches, only minor variation with cutoff threshold can be expected due to the effect of the different set choice for each experiment on the subsequent normalization. Signatures 1–11 seemed to be particularly stable.

The question arises whether even more data could have been removed without affecting the stability of the signatures of high relative data power. Removing 35% or even 50% of the original variables actually has similar results in that signatures with higher relative data power are more likely to be conserved. In each case, two-thirds of the signatures could be identified in all six pairwise comparisons, and they all had a good score (data not shown).

So, do these numbers improve if less data is removed? Interestingly, there is no such trend (data not shown). Rather, it seems that removing small

Copyright © 2003 John Wiley & Sons, Ltd. 

**Table 5.** Set 1 signatures identified in all pairwise matches to signature sets obtained after randomly excluding entire measurements, for different thresholds

| *p* (%) | Cutoff | *N* | Identified signatures with good (ok) similarity scores |
|---|---|---|---|
| 50 | $2.5 \times 10^{-3}$ | 30 | (1) (2) (3) (6) (15) (16) (21) |
| 50 | $3.6 \times 10^{-3}$ | 24 | (1) 2 (4) (6) (15) |
| 50 | $4.3 \times 10^{-3}$ | 18 | 2 (3) (15) |
| 50 | $7.5 \times 10^{-3}$ | 12 | 2 (9) (11) |
| 65 | $2.0 \times 10^{-3}$ | 36 | 1 3 (4) (5) 6 (7) (8) (9) (11) (16) (20) (22) (23) (26) 31 |
| 65 | $2.5 \times 10^{-3}$ | 30 | 1 3 (5) 6 (9) (11) (14) (19) (20) (21) (22) (26) (28) (30) |
| 65 | $3.6 \times 10^{-3}$ | 24 | 3 (5) 6 (8) (9) (10) 11 |
| 65 | $4.3 \times 10^{-3}$ | 18 | 1 3 (4) (10) 11 (12) |
| 65 | $7.5 \times 10^{-3}$ | 12 | 1 3 (4) 6 (7) (8) (9) 11 |
| 80 | $2.0 \times 10^{-4}$ | 48 | 1 2 4 (5) 6 8 (9) (10) 19 (22) 25 (27) 28 (29) 30 31 (32) 36 37 43 (46) |
| 80 | $1.2 \times 10^{-3}$ | 42 | 1 2 (3) 4 (5) 6 8 (9) (10) (14) (16) 19 (20) (22) 25 28 (29) 30 31 (32) 36 37 |
| 80 | $2.0 \times 10^{-3}$ | 36 | 1 2 (3) 4 6 (7) 8 (9) (13) (14) (22) 25 (26) 28 (29) 30 (31) (32) (33) 36 |
| 80 | $2.5 \times 10^{-3}$ | 30 | 1 2 (3) 4 (5) 6 (7) 8 (9) (14) (15) (16) (18) (20) (22) (23) 25 (27) (28) 30 |
| 80 | $3.6 \times 10^{-3}$ | 24 | 1 2 3 4 (5) 6 (9) (12) (14) (15) (21) (22) (23) |
| 80 | $4.3 \times 10^{-3}$ | 18 | 1 2 3 4 (5) 6 (12) (14) |
| 80 | $7.5 \times 10^{-3}$ | 12 | 1 (2) (3) 4 6 (7) 8 (9) |
| 95 | $1.2 \times 10^{-37}$ | 54 | 1 2 3 4 5 6 7 8 9 (10) (11) 12 13 16 18 (19) 20 21 22 (24) (25) 26 27 28 (30) 31 (33) 34 (35) 36 (39) 40 (41) (42) (43) 44 (45) 47 49 53 (54) |
| 95 | $2.0 \times 10^{-4}$ | 48 | 1 2 3 4 5 6 7 8 9 (10) (11) (12) 13 16 18 (19) 20 21 22 (23) (24) (25) 26 27 28 (29) 31 34 (35) 36 (38) (39) (41) 44 (45) 47 |
| 95 | $1.2 \times 10^{-3}$ | 42 | 1 2 3 4 5 6 7 8 9 (10) (11) (12) 13 (14) (15) 16 18 19 20 21 22 (23) (24) 26 27 28 (29) 31 (33) 34 (35) (36) (38) (39) (40) (41) |
| 95 | $2.0 \times 10^{-3}$ | 36 | 1 2 3 4 5 6 7 8 9 10 13 16 18 19 20 21 22 (23) (24) 26 27 28 (29) 31 (32) 34 |
| 95 | $2.5 \times 10^{-3}$ | 30 | 1 2 3 4 5 6 7 8 9 10 (11) (12) (13) (14) 16 (17) 18 20 21 22 (23) (24) 26 27 28 (29) |
| 95 | $3.6 \times 10^{-3}$ | 24 | 1 2 3 4 5 6 7 8 9 (10) (12) 13 14 16 (17) 18 (19) 21 (23) |
| 95 | $4.3 \times 10^{-3}$ | 18 | 1 2 3 4 5 6 7 8 9 (11) (12) 13 (15) 16 18 |
| 95 | $7.5 \times 10^{-3}$ | 12 | 1 3 4 5 6 8 |

ICA was performed for four sets of data, in each of which a percentage *p* of the original 63 measurements had been retained randomly. The signatures of an arbitrary set of ICA results (set 1) were then determined which could be identified in *all* the pairwise comparisons to the remaining three signature sets. This table lists these signatures for various relative data power thresholds, and also shows which of them had a good similarity score ($\geq$0.6). *N* gives the total number of signatures in the sets that were compared, i.e. this number of signatures were above the cutoff for all sets.

or even large amounts of the original variables has a similar impact on the signatures as any other minute changes, such as picking a different seed for the random number generator, essentially leaving between two-thirds and 90% of recoverable signatures, most of which have a good score. Apparently, the robust signatures found are characterized by many genes, so that dropping even a larger proportion of genes does not preclude picking up these signatures.

### Effects of excluding random subsets of measurement samples

Already Figure 13 shows that in a setting of relatively few measurement samples and many variables, removal of the latter has less serious consequences than removal of the former (cf. Figure 10). At high enough relative data power ($\sim$1%), however, many signatures can still be identified (Figures 14 and 15). At a threshold of 1%, in pairwise matches, about two-thirds of the signatures were always identified (half of which having a good score) after 20% of measurement samples had been removed from each set. When fewer samples are removed, this proportion increases significantly ($>$80% ok, two-thirds with good score), and it is much smaller when even more data is dropped. Only one-quarter of signatures could always be identified when half of the measurement samples had been removed, and less than 10% of signatures could be matched with a good score; cf. Table 5. The fact that by using a data set of only twice the size, we can reliably extract three times as many

Copyright © 2003 John Wiley & Sons, Ltd. 

signatures and match six times as many signatures with a good score highlights the importance of independent measurement samples in a situation where few such samples are available.

### Conclusion

ICA on yeast gene expression ratio data has proved to be fairly robust. The data set studied consisted of 63 measurements of expression ratios for 4870 genes. A relative data power threshold of ∼1% gave a set of 12 signatures, all of which could be clearly identified in analysis reruns with different random number generator seeds, with the occasional exception of one or two signatures, depending on the exact choice of the relative data power threshold. Even after random exclusion of substantial amounts of data, most of the original 12 signatures could clearly be identified. This was demonstrated by removal of e.g. up to 50% of the original input variables (particular gene expression ratios) or 20% of entire measurements (independent samples), after which most signatures could still be clearly identified, missing less than one-third of signatures, depending on the choice of relative data power threshold. When only 20% of the original input variables (particular gene expression ratios) had been removed, almost all signatures were matched reliably (i.e. with only one or two exceptions, depending on the choice of data power threshold).

In summary, the observed robust signatures were characterized by many genes, which made them fairly immune to even crude omissions from the data. Eventually, the 63 measurements yielded ∼10 reliably reproducible signatures. This is highly surprising and noteworthy in its own right, considering that the wild-type 'control' data has traditionally only been treated as noise. The signatures obtained are presently being studied for biological relevance.

This work should be repeated on independent data sets to see whether the recommended cut-off value of ≈1% relative data power also transfers to other analyses, e.g. gene classification studies by expression profiles over time (Hori *et al*., 2001, 2002). It can be expected, however, that at least another such threshold can be found.

### Online supplement

Further details of this study, including additional figures, can be found at **http://www.inference.phy.cam.ac.uk/is/papers/sup**

### Acknowledgements

We wish to thank Johan Rung (EMBL–EBI) for discussion and advice regarding preparation of the data sets used in this study. D. Kreil acknowledges support by a Medical Research Council Research Training Fellowship (G81/555). D. MacKay's group is supported by the Gatsby charitable foundation.

### References

Bell AJ, Sejnowski TJ. 1995. An information-maximization approach to blind separation and blind deconvolution. *Neur Comput* **7**(6): 1129–1159.

Bussemaker HJ, Li H, Siggia ED. 2001. Regulatory element detection using correlation with expression. *Nature Genet* **27**(2): 167–171.

Hori G, Inoue M, Ichi Nishimura S, Nakahara H. 2001. Blind gene classification based on ICA of microarray data. In *ICA2001: 3rd International Conference on Independent Component Analysis and Signal Separation*, vol. 3, Lee T-W, Jung T-P, Makeig S, Sejnowski TJ (eds). San Diego, CA; 332–336.

Hori G, Inoue M, Ichi Nishimura S, Nakahara H. 2002. *Blind Gene Classification — an ICA-based Gene Classification/Clustering Method.* Technical Report, Laboratory for Advanced Brain Signal Processing, Brain Science Institute, RIKEN, Saitama 351-0198, Japan.

Hughes TR, *et al*. 2000. Functional discovery via a compendium of expression profiles. *Cell* **102**(1): 109–126.

Hyvarinen A, Karhunen J, Oja E. 2001. *Independent Component Analysis*. Wiley: New York.

Liebermeister W. 2002. Linear modes of gene expression determined by independent component analysis. *Bioinformatics* **18**(1): 51–60.

Lin SM, Liao X, McConnell P, *et al*. 2002. Using functional genomic units to corroborate user experiments with the rosetta compendium. In *Microarray Data Analysis*, vol. 2, Lin SM, Johnson KF (eds). Kluwer Academic: Dordrecht; 99–103.

MacKay DJC. 1995. Developments in probabilistic modelling with neural networks — ensemble learning. In *Neural Networks: Artificial Intelligence and Industrial Applications. Proceedings of the 3rd Annual Symposium on Neural Networks, Nijmegen, The Netherlands, 14–15 September 1995*. Springer: Berlin; 191–198.

MacKay DJC. 1996. Maximum likelihood and covariant algorithms for independent component analysis. Technical report, Inference Group, Cavendish Laboratories, University of Cambridge. **http://www.inference.phy.cam.ac.uk/mackay/abstracts/ica.html**.

Makeig S, Jung TP, Bell AJ, Ghahremani D, Sejnowski TJ. 1997. Blind separation of auditory event-related brain responses into

Copyright © 2003 John Wiley & Sons, Ltd. 

independent components. *Proc Natl Acad Sci USA* **94**(20): 10 979–10 984.

Miskin JW. 2001. Ensemble Learning for Independent Component Analysis. PhD Thesis, University of Cambridge.

Pearlmutter BA, Parra LC. 1996. A context-sensitive generalization of ICA. In International Conference on Neural Information Processing, Hong Kong.

Roberts S, Everson R (eds). 2001. *Independent Component Analysis: Principles and Practice*. Cambridge University Press: Cambridge.

Schena M, Shalon D, Davis RW, Brown PO. 1995. Quantitative monitoring of gene expression patterns with a complementary DNA microarray. *Science* **270**(5235): 467–470.

Copyright © 2003 John Wiley & Sons, Ltd. 
